# Editorial: Mental Health Challenges in Elite Sport: Balancing Risk with Reward

**DOI:** 10.3389/fpsyg.2017.01892

**Published:** 2017-10-25

**Authors:** Tadhg E. MacIntyre, Marc Jones, Britton W. Brewer, Judy Van Raalte, Deirdre O'Shea, Paul J. McCarthy

**Affiliations:** ^1^Health Research Institute, University of Limerick, Limerick, Ireland; ^2^Centre for Sport, Health and Exercise Research, Staffordshire University, Stoke-on-Trent, United Kingdom; ^3^Department of Psychology, Springfield College, Springfield, IL, United States; ^4^Department of Personnel and Employment Relations, University of Limerick, Limerick, Ireland; ^5^School of Health and Life Sciences, Glasgow Caledonian University, Glasgow, United Kingdom

**Keywords:** mental health, sport psychology, stigma, psychological literacy, green exercise, depression, sport injury

Mental health is a global societal challenge. Sport, more specifically, elite sport, offers a potential window into the mental health challenges of young people. We initiated this research topic by proposing that explanations for mental health disturbance in sport predominantly based on training load (e.g., mental health model, Raglin, 2001), overlooked the potential organizational stressors in the high performance sport environment and did not adequately account for sport-related issues, including the consequences of injury, for non-normative transitions out of sport (Brewer and Redmond, 2017). In parallel with articles published in this research topic, recent research has advanced our knowledge of the prevalence of psychological disorders in elite sport, highlighting mental health issues among elite sport performers (e.g., Rice et al., 2016; Gouttebarge et al., 2017; Hagiwara et al., 2017). In light of this contemporary research, the present research topic on “Mental health challenges in elite sport: Balancing risk with reward” in Frontiers in Psychology makes a contribution with 17 articles comprising original research, reviews, perspective features, and an abundance of commentaries on this important topic. Depression was a foremost concern highlighted in the articles included in this research topic, which was investigated with novel methodological approaches.

Three papers considered depression amongst athletes and sports performers. Newman, Howells, and Fletcher considered the depressive experiences of elite sports performers using autobiographical approaches, a narrative approach adopted previously in resilience research (Morgan et al., 2015). Beyond the novel method, the article opened up new avenues of insight regarding the temporal and reciprocal relationship between depression and sports performance. The findings demonstrated that the reciprocal relationship between depression and sports performance changes over time; initially while sport may offer an escape from depressive symptoms, over time the demanding nature of sports can shift the relationship from facilitative to debilitative. This highlights the importance of considering both positive and negative aspects of sports engagement, particularly if success in sports is relied upon for increasing one's self-worth. An insightful paper by Males reinforced the caution expressed by Newman et al. that any analysis of retrospective accounts particularly, with a ghost writer, may lead to an embellished narrative. Furthermore, Males notes that practitioners are often faced with a dilemma in dealing with performers; should they focus primarily on performance enhancement or should well-being be the priority? This issue for consultants has ramifications for the training of practitioners, raising the question as to whether models of training should encompass counseling skills.

A similar qualitative approach by Doherty et al. provides insights into the experience of depression during the careers of elite male athletes. Their findings reveal the link between an overly strong athletic identity, the public evaluation of performance, and an almost obsessive drive and will to win. The findings also demonstrate the less than helpful masculine environment of elite sport in contributing to depression. The experience of depression among males was not dissimilar to the manifestation of depression in men more generally, including feeling of failure, shame, global negative self-evaluation, and a perception that depression was not acceptable. More specific to their environment, it was also associated with overtraining and efforts to display mental toughness. Finally, this paper shed light on some of the maladaptive and adaptive processes of recovery. Overtraining to the extent that it could be considered self-harm appeared as a maladaptive coping strategy, while accepting and expressing one's real self, and broadening one's self of identity beyond the sports domain emerged as adaptive coping responses. In the commentary by Ringland, the “primacy of performance over the person” in elite sport systems was highlighted and the sporting vernacular which includes terms like “mental toughness” is a barrier to disclosure and may inhibit help seeking behavior (Bauman, 2016). Engaging multi-disciplinary support teams in supporting athletes well-being and preventing mental health in an integrated fashion was an interesting suggestion that would resonate across this topic. Arvinen-Barrow in their commentary reiterated the idea that professionals working in elite sport need to know their athletes and appreciate the triggers of psychological distress. Furthermore, this authors calls for more research from a biopsychosocial perspective with the entourage (i.e., everyone who has an influence on an athletes' performance—on and off the field of play).

Nixdorf et al. sought to understand the higher propensity to depression in individual sports compared to team sports. The findings of their cross-sectional study with almost 200 participants revealed that a negative attribution of failure mediated the relationship between individual sports and depression, but interestingly team cohesion showed no relationship. Elbe and Nylandsted-Jensen echo the conclusions of this article on the role of negative perfectionism in predisposing young athletes to depression. They highlight that the field of sport psychology beginning to address mental health through initiatives including position statements on mental health in sport (MHS) (Schinke et al., 2017). Longitudinal studies are recommended as a pathway to elucidating the causal factors of depression in sport and appraisal should be among the variables subject to measurement (Cumming et al., 2016).

Sport-injury was another topic among the contributions to the research topic. Our understanding of both the prevalence of sport injuries and their consequences have only recently come to the fore in elite sport settings. Most research to date has focused on the impact of injury on an individual player, but the impact of injury as a form of player attrition on remaining players in a team has received less attention. Hurley reflects on the 2015 Rugby World cup, suggesting that support staff may play a key role in helping teams cope with injury to key players, avoiding emotional contagion and the commensurate risk to performance stability.

Hill et al. examine the role of mental health and clinical issues within talent development, particularly focusing on young athletes and the need for early intervention. They conducted qualitative interviews with eight clinicians. Among the findings from a thematic analysis was that protective factors were social in nature, and risk factors include a lack of understanding and awareness of clinical issues. Their findings identified a distinct need for improved identification and interventions strategies, particularly amongst coaches.

These papers identified a number of limitations and shortcomings in the way in which mental health issues have been researched and addressed in practice. To comprehensively examine this, Uphill et al. provide a critical review of the manner in which mental health has traditionally been examined in sports players. They note the predominance of research which has focused on the language of mental illness, and how this has contributed to stigmatization and a reluctance on the part of athletes to seek help when needed. They draw on Keyes's (2002) two continuum model, which conceptualizes mental health and mental illness as two distinct continua (rather than two ends of a single continuum) to demonstrate that refocusing on mental health as a conceptual space that encapsulates both distressing and flourishing experiences can go some way toward alleviating the stigma traditionally associated with mental health. This review presents an important benchmark to the field regarding the way in which the approach to mental health issues should change going forward. Uphill et al. also comment on the ways in which we can intervene to either reduce the prevent mental distress, or to develop and protect flourishing. A similar viewpoint is expressed by Roberts et al. in their reflection on the relationships between performance enhancement and common mental disorders in the UK athletic population. They advocate that the field of applied sport psychology need to evolve to ensure that it continues to meet the demands of its clients beyond issues of performance enhancement.

An alternative account is proposed by Lebrun and Collins who question the appropriateness of applying screening tools with athletic samples when the cut-offs are extrapolated from non-sporting populations. They propose that until causal findings are established, sport should be considered as an achievement context where functionality or non-functionality should be the criteria for intervention. Recent findings in the UK from the Duty of Care report (Department for Digital Culture Media Sport, 2017) suggest that maladaptive behaviors are commonplace even in functional sport systems, and that athletes mental health is under threat. To intervene or not is a question that will promote further discourse in the future.

The latter articles in this research topic focus on the effectiveness of different types of interventions to address mental health. For instance, Turner's article provides a review of Rational Emotive Behavior Therapy in addressing the former, and specifically for reducing irrational beliefs and increasing rational beliefs, and associated emotions.

While much research focuses on the individual sports performer, contextual factors are also important to consider, and these may present a point for intervention. In particular, the knowledge and openness of support staff may be a key mitigating factor regarding the alleviation of mental health stigma in sports. Sebbens et al. consider the role of knowledge and confidence of elite sport staff to enhance early intervention. They delivered a 4 h MHS workshop to coaches and support staff. Their results showed that participants increased their knowledge of the signs and symptoms of common mental illnesses and had higher self-efficacy to help someone who may be experiencing mental health problems. Gulliver commends the effectiveness of mental health training programmes but poses questions for the role of coaches at the nexus of the performance domain in sport. Is mental health stigma an issue for them and is mental health part of their playbook or do they divest themselves of this responsibility. Sharing the responsibility for mental health awareness and positive actions across multi-disciplinary teams is integral to creating a supportive and flourishing environment.

The broader environmental context is the subject of the article by Donnelly et al. and an accompanying commentary by Rogerson. A novel approach is outlined in which the environmental risks and possible benefits of natural spaces are outlined using evidence from recent Olympic games venues. The authors consider environmental influences of green spaces on mental health and well-being which moves the focus away from mitigating environmental hazards (e.g., air pollution) to enhancing protective factors. Rogerson's commentary on this article additionally points to the potential ergogenic value of natural environments relative to competition in a built environment. This raises the question of whether the Nike *breaking 2* project would have been achieved had the venue been the Michael Johnson track situated in a pine forest in Oregon rather than the built environment of the Monza F1 racing track in Italy. Evidence to support the role of nature in providing a boost to well-being is emerging (Lawton et al., 2017).

Understanding mental health is a key precursor to the development of interventions and strategies to enhance mental health within the sports domain. Taken as a whole, two key messages resonated from the articles included in this research topic. Firstly, mental health stigma is a barrier to disclosure, self-helping behavior and support for those with psychological distress. It has been acknowledged that stigma can lead to under-reporting and service aversion (Gulliver et al., 2012). Secondly, recognition of mental health status is not simply an issue for sport psychologists and their clients, but a shared mandate within sport systems is necessary for both mental health prevention and the promotion of well-being.

The implications of the aforementioned themes are 2-fold. Firstly, we advocate exploratory research to understand the barriers to reporting among athletes, coaches, and other members of the athletes' entourage. Future studies should augment prototypical approaches to mental health surveys with measures of mental health stigma, help seeking behaviors, attitudes toward service provision and barriers and enablers to reporting an issue.

A further recommendation for future research is how to conceptualize mental health awareness. To date, it has largely been viewed in terms of mental health literacy (e.g., mental health first aid) and mental welfare (Department for Digital Culture Media Sport, 2017). Both these terms set the bar low in terms of what cognitive and meta-cognitive knowledge can be shared to enhance mental health and well-being. We should be promoting a broader concept of psychological literacy to stakeholders in sport, one that promotes well-being for all, providing a toolkit for autonomous regulation of athletes psychological resources. Interestingly, few studies have even explored the concept of *thriving* within sport systems (Brown et al., in press) and this offers a fruitful pathway for future research. Exploring conceptualisations of well-being among athletes, coaches, and other members of the entourage would be useful in assessing the possibility of promoting the dual-axes model of mental health (Keyes, 2002) within the elite sporting landscape.

This research topic has contributed to the extant literature, not just answering longstanding questions but by posing new questions to advance the research conceptually, methodologically and finally in terms of practice impact for enhancement of the lives of elite athletes and their entourage in the future.

## Author contributions

DO created an early draft of Editorial which was then expanded upon by TM and final edits and approval was given by the remainder of the authors.

### Conflict of interest statement

The authors declare that the research was conducted in the absence of any commercial or financial relationships that could be construed as a potential conflict of interest.
